# High Salt Diet Affects the Reproductive Health in Animals: An Overview

**DOI:** 10.3390/ani10040590

**Published:** 2020-03-31

**Authors:** Sameh A. Abdelnour, Mohamed E. Abd El-Hack, Ahmed E. Noreldin, Gaber Elsaber Batiha, Amani Magdy Beshbishy, Husein Ohran, Asmaa F. Khafaga, Sarah I. Othman, Ahmed A. Allam, Ayman A. Swelum

**Affiliations:** 1 Department of Animal Production, Faculty of Agriculture, Zagazig University, Zagazig 44511, Egypt; samehtimor86@gmail.com; 2 Department of Poultry, Faculty of Agriculture, Zagazig University, Zagazig 44511, Egypt; 3 Histology and Cytology Department, Faculty of Veterinary Medicine, Damanhour University, Damanhour 22511, Egypt; nourislam2010@yahoo.com; 4 National Research Center for Protozoan Diseases, Obihiro University of Agriculture and Veterinary Medicine, Nishi 2-13, Inada-cho, 080-8555, Obihiro, Hokkaido, Japan; gaberbatiha@gmail.com (G.E.B.); amanimagdi2008@gmail.com (A.M.B.); 5 Department of Pharmacology and Therapeutics, Faculty of Veterinary Medicine, Damanhour University, Damanhour 22511, AlBeheira, Egypt; 6 Department of Physiology, Veterinary Faculty, University of Sarajevo, Zmaja od Bosne 90, 71 000 Sarajevo, Bosnia and Herzegovina; husein.ohran@vfs.unsa.ba; 7 Department of Pathology, Faculty of Veterinary Medicine, Alexandria University, Edfina 22758, Egypt; Asmaa.Khafaga@alexu.edu.eg; 8 Biology Department, Faculty of Science, Princess Nourah bint Abdulrahman University, Riyadh 84428, Saudi Arabia; sialothman@pnu.edu.sa; 9 Department of Zoology, Faculty of Science, Beni-suef University, Beni-suef, 65211 Egypt; allam1081981@yahoo.com; 10 Department of Animal Production, College of Food and Agriculture Sciences, King Saud University, P.O. Box 2460, Riyadh 11451, Saudi Arabia (AAS); aswelum@ksu.edu.sa; 11 Department of Theriogenology, Faculty of Veterinary Medicine, Zagazig University, Zagazig 44511, Egypt

**Keywords:** high salt intake, reproductive health, animals, sperm function, ovarian follicles

## Abstract

**Simple summary:**

Halophytic plants are a promising animal feed source. However, the extreme NaCl_2_ salt content constraints their use. Excess diet salt adversely affects growth performance and animal’s reproduction worldwide. This review focuses on the impact of high salt intake on growth performance and reproduction ability in animals.

**Abstract:**

Salinity is a reliable issue of crop productivity loss in the world and in certain tropical and subtropical zones. However, tremendous progress in the genetic improvement of plants for salinity tolerance has been made over several decades. In light of this, halophytic plants can be used as animal feeds and have promising features because they are a good feed resource. However, the main constraint of saline pasture systems is the extreme concentration of NaCl salt in drinking water and forage plants for grazing animals. Ecological reports revealed that excess diet salt causes mortality and morbidity worldwide. Animal fed halophytic forages may have adverse effects on growth performance and reproductive function in males and females due to inducing reductions in hormone regulation, such as testosterone, FSH, LH, and leptin. It was indicated that high salt intake promotes circulating inflammatory factors in the placenta and is associated with adversative effects on pregnancy. This review focuses on the scientific evidence related to the effect of high salt intake on growth performance, spermatogenesis, sperm function, and testicular morphology changes in male animals. In addition, the review will also focus on its effect on some female reproductive features (e.g., ovarian follicle developments, placental indices, and granulosa cell function).

## 1. Introduction

With the rise of population in the world and decrease in the use of lands for agriculture practices, several approaches in agriculture systems involve the culture of plants in salinity land for animal feeding [1,2]. Moreover, many countries are suffering from scarcity of freshwater with an increase in the ambient temperature which had led to a decrease in the availability of freshwater. Recently, genetic scientists in the field of plants have produced some genetically modified plants, which could be able to grow in the salinity land. However, the main drawback of saline pasture systems is having the high salinity of drinking water and forage plants for grazing animals. Studies reported that the salt content of some forages such as *Suaeda glauca* has up to 317–331 g/kg dry matter (DM), while numerous other forages contain 15–41 g/kg DM with higher than the salt content of 1.2–1.8 g/kg DM in most forages [3]. Furthermore, surface water in saline regions comprises up to 0.9–1.7% salt, which leads to an exacerbation of the enhanced salt consumption [4]. Several types of animal disorders have been observed such as lipid accumulation, kidney damage, hypertension, and failure in renal function as a result of an excess intake of salt [5]. Moreover, nowadays, there is an increasing proof to examine the relation between high salt consumption and many disorders that include obesity, water retention, osteoporosis, cancer, obesity, vascular dementia, and diabetes [6,7,8]. Recently, increasing evidence revealed that a salt-rich diet stimulates the cytokine interleukine-17 in the gut, which leads to aberrant brain microvasculature, changed cognitive injury, and brain function [9]. Aguiar, et al. [10] indicated that high salt causes inflammation in mice’s colonic mucosa through increased incidences of type 3 innate lymphoid cells (ILC3) and IL-23R+ CD4 + T cells and was reliant on interleukin-17 (IL-17). Moreover, the high prevalence of cardiovascular diseases has been related to excessive processed meat ingestion, partly due to higher salt intake. In vivo experiments have shown that a salt-rich diet possesses a negative impact on intestinal health, by inducing autoimmune disorders and tissue dysfunction [11].

Infertility potentially implicates several issues, but this review focuses mostly on the influences of high salt on the reproductive function in animals. Regarding the female animals, high salinity suppressed the recruitment and development of ovarian follicles, thereby increasing infertility [7]. Additionally, placental changes suggest that even modest dietary intakes of usually “Western” diet constituents can alter placental functions in ways that demonstrate placental failure [12]. Additionally, researches have recognized that high intake of salt has harmful effects irrespective of the increase in blood pressure, hypertension, and cardiovascular diseases [13,14]. According to previous facts, health associations have issued guidelines to minimize the consumption of salt [15,16]. Nevertheless, experimental reports did not explicitly indicate a health gain from such treatments, which posed concerns about the scientific data that supports the random limit of salt in the overall population [17,18]. Consequently, there has been a longstanding debate regarding the efficacy of reducing sodium intake and a call for further research to obtain a deeper understanding of its health and syndrome properties [19].

The examination of reproductive health in animals fed a high-salt diet revealed an impairment in sperm function, disturbances in hormonal regulation, reduced testicular weight, and alterations in testicular morphology and gene expression related to sperm quality in males [3]. Additionally, previous reports concluded that intake of halophytic forages might have deleterious influences on growth traits [3,20], milk production, and fetal development [4], through direct or indirect effects [3,21,22]. High salt intake disrupts the normal reproductive function in animals, which may manifest as decreased testes weight, sperm function, and sexual hormones in rams [3], and alters molecular transcripts for some related genes. High salt in water has been shown to decrease reproductive efficiency in relation to diets in males and young animals in contrast to females and adult animals.

This review intended to illustrate related studies concerning the possible hazards of dietary high-salt on growth rates, hormonal homeostasis, testicular morphology, sperm function, gene alteration related to spermatogenesis and maturation, and in vitro fertilization results in different males of animal species; furthermore, it summarizes the negative influences of high salt diets on female reproductive features, such as ovarian follicle developments, hormonal profile, placental indices, and granulosa function.

## 2. Growth Performance

Studies on animals revealed that high salt reduces the growth performance parameters: live body weight gain (LBWG), daily body weight gain (DBWG), and feed and water intake (FI and WI, respectively), and decrease the digestibility of nutrients [3,20,23,24]. On the other hand, some reports indicated that high salt consumption had no effects on LBWG, DBWG, and FI and WI) [25,26,27]. Rams exposed to 12% salt for three months had lower LBWG (*p* < 0.05) and higher WI (*p* < 0.05) relative to normal salt diet (4%) [3]. In the study of Fang et al. [3], authors selected this level of salt based on the contents of salt in some forages which are normally grown on salt-tolerant grass, comprising *Puccinellia tenuiflora*, *Leymus chinensis*, Chloris, and *Suaeda glauca*, in Jilin province’s western grassland [3]. The consumption of high salt diets resulted in lower growth performance in sheep [3], whereas LBWG was slower relative to high salt content [20]. One explanation for the disturbances in animal performance raised in the salt-land pasture has frequently been attributed to reduced FI and poor plant digestibility [23], it is more probable owing to excess sodium intake [28]. Results indicated that the growth performance of animals reared in saline land pasture would be decreased, while halophytic shrubs only accounted for 30% of the total pasture diet. In Sprague–Dawley rats, dietary high salt (8% salt) decreased significantly the FI and LBWG compared with the standard chow group [24]. The specific effect of high salt consumption on FI and LBWG in most animals in the experiments is controversial. Ogihara et al. [27] found that feeding rats with high salts had no significant effects on BWG and FI. The feature accountable for this contrasting statement between FI and LBWG is not clear, but could be associated with alterations in the metabolism of nutrients [24]. Wang et al. [25] indicated that consumption of high-salt diet did not exhibit drastic alterations in FI, LBWG, and the altitudes of numerous inflammatory issues. However, they observed that excess salt in the diet could hinder the host’s digestive enzyme excretion, alter the biological process, the molecular function of the duodenal content, and the cell constituent, and further alter the composition of the intestinal microbiota [25]. In addition, Lins et al. [26] examined the effects of different water salinity doses (640, 3188, 5740, and 8326 mg/L) on the growth performance of prepubertal male sheep. They reported that water salinity did not affect water intake or BW. Wang et al. [7] found that female mice that received high salinity water (4% NaCl) exhibited a significant reduction in feed intake and water consumption compared with the control mice. Female mice exposed to high salinity in water (4%) showed a significant reduction in growth performance [7]. These discrepancies in growth might be related to reducing feed and water intake as a result of high salinity exposure. Feed intake was markedly decreased in cattle and sheep when dietary salt levels exceeded 10%, with a decrease in digestibility in sheep at 15% salt. The WI was increased to 4 liters when 100 g of salt were ingested by sheep and cattle. The exact molecular machinery which explains the negative influences of high salt diet on the growth performance remains unclear. High salt intake could result in alterations in some enzymes related to protein digestion and cause an upregulation of fatty acid oxidation [29]. Gut microbiota plays a major part in immunological and nutritional processes in the digestive canal [25], and changes in the composition of microbiota in the gut can affect animals and human well-being and health [30]. High salt in the diet might lead to an increase in the lipid deposition in tissues, increased lipid-metabolic enzyme expression, and glutamate metabolic pathways [31]. Scarce data on the effect of the high-salt diet on genetic changes in the digestive intestine of microbiota are nevertheless accessible. More explorations and molecular mechanisms to illustrate the undesirable effects on the performance are required.

## 3. Reproductive Variables in Males

### 3.1. Reproductive Organ Weight

It is accepted that sperm synthesis and function are positively linked to testis weights and size, since larger testicles have a greater sperm mass. So, test size or weight might represent a possible indication of the number of sperms that will be produced. A critical factor that affected testes size certainly has direct effects on sperm production. A testis weight decrease by 22.8% (*p* < 0.05) was achieved in rams whose feed was high salt diets (12%) compared with those that had 0.5% salt, however, they had lower values for spermatogenic index 4 and 5, but higher values for 2 and 3 (*p* < 0.05) [3]. In mice, Wube, et al. [32] noticed that 3.5% or 5% NaCl for 6–8 wk decreased both spermatogenesis and testis weight. Feeding rats high salt resulted in decreased testis weights (*p* < 0.05), seminal vesicle weight, and alterations in testicular mass, which may reveal changes in seminiferous tubules [33], and finally a change in the quality and quantity of sperm [24]. This is evidence that the energetic role of testicular morphology and seminal vesicular secretion are vital for sperm function and stability of sperm chromatin [34]. Additionally, the weight of seminal vesicles is joined with the other organ weights and its secretion [35]. Hence, the lessening in weight detected possibly reflects the reduced secretions of the seminal vesicle. In prepubertal male sheep, Lins et al. [26] mentioned that water salinity at different levels had no significant effect on scrotal indices. High salt in diet or water could reduce the spermatogenesis and alter the testicular morphology leading to reduced fertility in male animals.

### 3.2. Sperm Function

Sperm function as revealed by sperm concentration, viability, morphology, and motility is an essential measure of male fertility competence, while the quality of sperm is associated with low fertility ability [36]. In rams, Fang et al. [3] reported that animals fed 12% salt had a decreased ejaculate volume and sperm concentration and an increased DNA fragmentation rate (*p* < 0.05) by 28.2, 35.8, and 417% compared with those in the control group (0.5% NaCl). Adverse impacts of high salt on rams have been reported to reduce testis size and alter spermatogenic index, leading to decreased ejaculate volumes, reducing sperm count per ejaculate, and higher DNA fragmentation [3]. In rats, Adekunbi et al. [24] reported a significant decrease in epididymal sperm concentration, motility, and viability (*p* < 0.05), as a result of high diet salt (8%) compared with the control condition. In addition, a significantly high percentage of abnormalities (*p* < 0.05) was detected in treated rats (8 per cent high salt diet) relative to control rats. Another study observed that rats fed the diet with high salt (8%) had a significant increase (*p* < 0.05) in sperm abnormalities, while no significant effects were detected in sperm motility with high or low salt diet [37].

Unexpected results have been obtained by Lins et al. [26], who demonstrated that the moderate level of salt in drinking water of prepubertal male sheep exhibited beneficial influences on sperm function such as sperm motility, concentration, and vigor. However, higher abnormalities were observed in this study. This function is possibly due to the existence of water salinity. Ca^2+^ is responsible for the polarization and depolarization of the myofibrils of flagella, which can lead to sperm movement [26]. Moreover, these ions are imperative for sperm motility, metabolism, acrosome reaction, hyper activated motility, and fertilization [38]. Regarding apoptosis in tests, Lins et al. [26] detected a higher percentage of apoptotic spermatogonia and early spermatids when male sheep were fed with high water salinity (8326 mg/L), indicating defects in spermatogenesis. High salt intake probably increases oxygen species in cells of the renal medulla of male hamsters [39], and viable equine sperm [40], leading to promote DNA damage in the cells.

Animal studies have confirmed that suitable supplementation of minerals can improve reproductive efficiency. When there is excess salt in the diet, it causes disturbances in the mineral homeostasis in body fluids, leading to alterations in the body functions. Also, an increase in salt diets could promote infertility, accumulate lipids, liver, and renal diseases and disorders. Regarding the impacts of high salt diet on mineral profile, Adekunbi et al. [24] concluded that the serum content of chloride ion was significantly increased in rats fed high salt (8 percent) without any changes in other electrolytes being measured. Similarly, an earlier study of Ofem, et al. [41] detected an elevation in chloride ion level and no alteration in sodium-ion contents after feeding on high salt diet. Contrary to previous results, high intake of salt diets will result in sodium retention and extracellular fluid volume expansion, thereby elevating plasma sodium content. Morita, et al. [42] also stated that a high intake of salt did not result in major alterations in serum electrolyte levels but increased sodium excretion in the urine.

### 3.3. Hormones

Mechanisms of high salt intake on hormonal regulation have been illustrated in several studies. Significant reductions of leptin, testosterone (T), insulin, luteinizing hormone (LH), and follicle-stimulating hormone (FSH) levels were observed by feeding rams with high salt diet (12 percent) relative to controls (*p* < 0.05) [3]. Leptin is well known to have a critical function in signaling nutritional status in the mammal’s centrally reproductive axis and tends to at least be a critical factor for puberty initiation, as it induces GnRH and LH releases [43]. The reduction of leptin and insulin might be attributed to the lowered body mass which is accompanied by a decrease in fat reserves. These hormones also play a major role in metabolism regulation, homeostasis, and body mass. Furthermore, the 20% salt diet of sheep reduced the peripheral level of insulin and the energy metabolism [44]. It was reported that high salt consumption declines both leptin and insulin content, independently of changes in FI or BWG [20]. Several previous studies have documented endocrine disorders in animals supplied with high salt diet, where significant decreases in plasma concentration of FSH, LH, and T were detected [3,45]. Regarding corticosterone, it was observed a substantial rise in corticosterone content and a remarkable reduction in the content of testosterone in rats, as influence by a high diet of salt was observed in the control group (0.5% salt) [24]. On the other hand, Adekunbi et al. [24] suggested that LH and FSH levels have not been affected by increased salt in the diet (8%) compared with control rats (0.5%). The reduction in testosterone production caused by a high salt diet might result in a reduction in spermatogenesis and alteration in sperm quality. In addition, the Leydig cell-synthesized testosterone was found to display no significant change in gonadotropin level in response to LH stimulation despite the low testosterone level. The high salt acted against the hypothalamic–pituitary axis by interacting with LH receptors in the Leydig cells [24]. Likewise, it has been indicated that high sodium consumption enhances the secretions of glucocorticoid hormones [46] by enzyme 11betahydroxysteroid dehydrogenase type 1 (11β-HSD1) [47]. It is well established that nutritional stress results in a high corticosterone level. Moreover, high levels of cortisol can reduce fertility [48], thus high sodium intake affects the reproductive function. Iranloye et al. [37] documented a significant improvement in FSH and testosterone levels when rat were fed with a high salt diet, however, a remarkable decrease in LH was observed when compared with controls (*p* < 0.05). This issue still needs to be investigated. Generally, a high salt diet induces a reduction in the body’s fat reserves, which leads to a decrease in the cholesterol content used for the synthesis of sex hormones. Thus, a reduction in the synthesis of sex hormones such as testosterone led to decreased spermatogenesis which contributes to reduced reproductive capacity.

### 3.4. Antioxidant Indices

Oxidative stress is accompanied by sperm malfunction and low fertility in animals [49,50]. Malondialdehyde (MDA) is considered a critical indicator of lipid peroxidation in the cells. Mice feeding on diets with high salt (8%) shown a marked rise in MDA level and superoxide dismutase (SOD) activities in the testis compared with controls [24]. GSH and catalase activities did not significantly differ between the high salt and control groups [24]. Similar to previous studies, Iranloye et al. [37] indicated that rats that received NaCl at 8% exhibited a relatively high MDA level in the epididymis compared to the controls. The activities of SOD, GSH, and CAT enzymes in testicular and epididymal tissues were significantly reduced by increased dietary salt in rats [37]. The high levels of MDA and SOD in the testis of rats fed a high salt diet can be attributed to free radical harm, confirming the harmful influences of the high salt intake on the reproductive patterns. Antioxidant enzymes are produced frequently in response to increased free radical concentrations; however, if antioxidant enzymes are disrupted by a high oxidative stress level, they can have harmful effects on reproduction.

### 3.5. Gene Expression

Studying the molecular mechanism of some genes related to reproduction reflects the negative impacts of high salt on sperm spermatogenesis and function. The key genes of spermatogenesis (Hsp70, c-kit, and Cyclin A) were reduced markedly (*p* < 0.05) by increased salt in the ram diet (12%), and decreased expression of sex hormone receptors, such as FSHR, AR, LHR, CYP17A1, and CYP11A1 in rams that received high salt levels [3]. However, no significant changes were observed between groups in testis key enzymes (ATPase, LDH, SDH, and AKP) [3].

Testis renin-angiotensin system (RAS) is receptive to different types of stresses and pathological conditions [51], suggesting that a disturbance of RAS may affect sperm functions and disturb the spermatogenesis process [52]. In the testis, a diet rich in salt diminished the REN’s transcriptional expression; hence, local downregulation of the synthesis of AngII which reduced anions as well as fluid secretion by testis, reflecting on testis function [53]. The mechanisms responsible for the changes in reproductive health due to excessive salt intake are still not completely defined. Therefore, more studies at molecular, transcriptomics, and proteomics analyses are needed to deeply understand the negative impacts of a high salt diet on sperm functions in male animals and humans.

**Figure 1:**
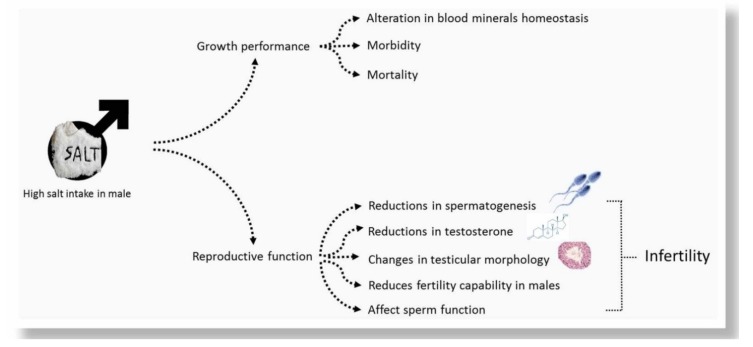
The adverse effect of high salt diet on male animals.

One explanation clarifying the changes during spermatogenesis due to a high salt diet is that it has great impacts on numerous key enzymes, such as AKP, SDH, LDH, and ATPase associated with cell proliferation and germ epithelium differentiation [54]. On the other hand, Fang et al. [3] suggested that dietary high salt had no effects on the abundance of ram testis enzymes. A high-salt diet therefore decreased the expression of main spermatogenic genes by decreasing the hormone reaction linked to spermatogenesis. Additionally, Ramaswamy and Weinbauer [55] clarified that the downregulation of the main spermatogenesis genes can be caused by Leydig cell loss or injury. The adverse effect of high salt diet on male animals is illustrated in Figure 1.

## 4. Female Reproductive Function

### 4.1. Ovarian Follicles

The histological examination of ovaries during the development of follicles reflects the negative impacts of high salt on fertility. Female mice exposed to high salt intake (4% in drinking water) had a fewer number of follicles at different phases than those in the control condition [7]. Moreover, a large number of atretic follicles and less corpus luteum were also found in salt-treated mice [7]. Overall, high salinity in drinking water or diet produces a fewer number in developing follicles and oocytes, and thus induce infertility in females.

### 4.2. Placental Indices

The placenta is an organ developed in the uterus throughout pregnancy. It plays a critical role in providing oxygen and nutrients to embryos and eliminates waste products from the embryo’s blood. Also, it is considered as an endocrine function organ, because of its ability to secrete some hormones related to maintaining pregnancy in some animals. Interruption of placental architecture throughout pregnancy is a relevant issue in prenatal development restraint and is also a characteristic of pregnancy syndromes like preeclampsia [56]. Recently, Reynolds et al. [57] indicated that maternal consumption of salt during pregnancy modifies FI and parental metabolism together with evidence of adverse effects on meta-inflammatory forms, insulin sensitivity, and weanling offspring adiposity. Parental high salt diet induced upregulation of inflammatory cytokines such as TNFα and IL-1β together with CD68 macrophage marker in the placenta [12]. There is recent evidence suggesting that high salt consumption stimulates inflammation factors in the placenta and is related to negative effects on pregnancy via disturbance of nutrient transport to the fetus [58]. Collectively, high salt consumption through the pregnancy period alters the placental morphological patterns and induces the inflammatory response leading to a decrease in provided nutrients to the embryo development and indicative of placental insufficiency.

### 4.3. Granulosa Cells

Differentiation and proliferation of granulosa cells (GCs) are critical for normal oocyte development, follicular growth, ovulation, and latinization [59]. High salinity is becoming an important environmental factor that has harmful influences on ovarian cells and consequently diminishes fertility is most mammal species [7]. Dysfunction of GCs due to any environmental or physiological issues leads to disordered ovarian follicle and oocyte development [60]. Phospho-histone H3 (PH3) is the main indicator as a biomarker for cell cycle and proliferation in stage G2 and mitosis (PH3 stains the condensed chromatin just before chromosomal segregation). Under high salinity, Wang et al. [7] detected less PH3+ granulosa cells in the follicles in comparison with the controls. Moreover, it has been reported that follicle-stimulating hormone receptor (FSH-R) protein in granulosa cells was suppressed under high salinity compared with a normal physiological range of salt [7]. High salinity exposure also decreased the abundance of PCNA, another measure of cell proliferation, while upregulated the cleaved caspase-3 [7]. Cumulative results indicated that one of the main explanations for the infertility phenomenon in mammalian species is the undesirable impact of high salinity on the growth and apoptosis of granulosa cells.

### 4.4. Hormones

**Figure 2:**
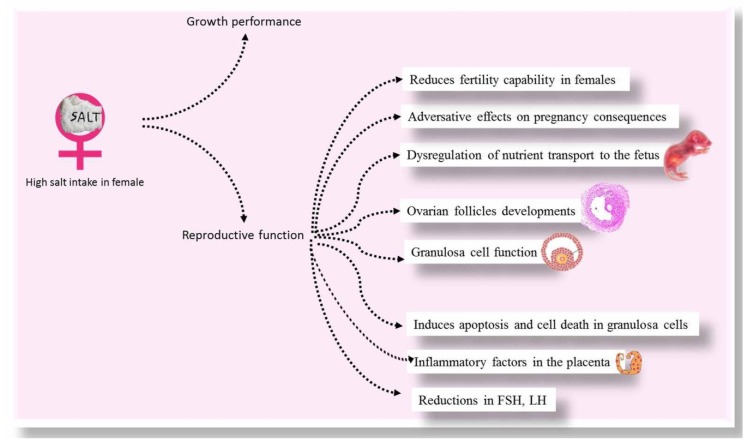
The adverse effect of high salt diet on female animals.

In female mice, the serum concentrations of progesterone, testosterone, and estradiol were not changed in mice fed with high salt-supplemented diets (4% in drinking water) compared with the control group [7]. However, high salinity is also reported to inhibit FSH secretion by the hypothalamus, which disrupts follicular development. The potential for high salinity to influence the role of the granulosa cells by controlling FSH-R expression remains to be studied in the future. Wang et al. [7] reported an elevation in sodium and chloride ions after exposure to high salinity water (4% in drinking water of female mice), representing its adverse effect on mineral balance in the body. The adverse effect of high salt diet on female animals is illustrated in Figure 2. Impacts of high salts on animals are briefed in Table 1.

## 5. Consequences of In Vitro Fertilization 

The female mice exposed to a diet with high salinity have been documented to be less fertile than the control ones [7]. This suggested that high salt treatment delays fertility during the mice’s growth period. For in vitro fertilization, it has been indicated that the cleavage rate was not affected by high diet salt in rams (12%), however, the hatching rate was lower by 70% in the 12%-salt group relative to the control one (*p* < 0.05) [3]. Increased DNA fragmentation show serious long-term effects on embryo development and offspring health [45], while having a diet with only 12 percent of salt did not alter the cleavage level, but had a negative impact on the hatching rate in sheep [3].

## 6. Conclusions

Generally, the use of halophytic plants in animal feeding is mostly associated with a reduction in reproductive performance and health problems. High salt intake may lead to decreased spermatogenesis, alterations in testicular morphology and declined spermatogenic function (concentration, viability, abnormalities, and motility). Furthermore, high salt diet reduces the synthesis of testosterone, FSH, LH, and leptin. Animal exposure to high salts increases the oxidative stress and induces the injury of the sperms and consequently reducing the reproductive performance in males. Additionally, studies on female animals revealed that high salt diet produces a smaller number of ovarian follicles, reduces cell proliferation, and induces apoptosis in granulosa cells. High salt intake also increases the circulating inflammatory factors that promote placenta inflammation and is linked to adverse effects of pregnancy by dysregulatory transport of nutrients to the fetus. Animal models are useful substitutes for humans in studies for both etiology and therapeutic interventions. Considering this, necessary steps should be provided, with the rise of salt intake in the western diet and takeaway foods, which might have detrimental effects on human fertility. The mechanism of negative impacts of high salt in western diets and fast foods in humans needs more investigations because it might be responsible for the low fertility nowadays.

## Figures and Tables

**Table 1:** Impacts of high salt diets on animals.

Items	Species	Dose	Main Findings	References
1- Growth performance	Sheep	(13% NaCl) diet during prenatal life	Modified thirst level, and blunted aldosterone reaction to oral salt levels.	[4]
	Sheep	80 g/kg DM	Reduced feed consumption, liveweight gain, digestion, and wool quality.	[20]
	Merino ewes	(14% NaCl) diet during prenatal life	Lower renin activity in lambs. The mineral content of the milk was changed. K concentrations were increased by 10 percent. Plasma Na of ewes has been reduced.	[21]
	C57BL/6J mice	3.15% NaCl	Improved the ratio of Firmicutes/Bacteroidetes and *Lachnospiraceae* and *Ruminococcus* abundances. Reduced *Lactobacillus* abundance. In the high-salt diet group, 20 low-abundance proteins were identified in duodenal content, which involved digestive enzymes and pancreatic secretion. No substantially differentiated proteins have been observed in colonic, cecal, and jejunal contents. There was an increased abundance of five proteins (trigger factor, undecaprenyl-diphosphatase, cytidylate kinase, transporter, and 6-phosphogluconate dehydrogenase). Two proteins were over-expressed including, PBSX phage manganese-containing catalase and acetyl glutamate kinase.	[25]
	Merino sheep	(20% NaCl of dry matter)	Eating large quantities of salt, reduce the amount of voluntary feed as well as the circulation of insulin and glucose concentrations. The high intakes of salt did not affect live weight and leptin concentrations in particular, but were reduced as a result of the decreased intake. Secretion of cortisol has not been affected. Although insulin and glucose have been affected by salt intakes (in addition to the effects of a decreased dietary intake), insulin intake is anticipated to decrease instead of increase consumption.	[44]
	Endothelin-1 knockout mice.	8% NaCl diet	High salt diet significantly improved the sodium excretion in urine and fractional excretion of sodium (FENa). The amount of circulating plasma, serum electrolytes, and creatinine clearance, or systemic blood pressure were not affected. Excretion of urinary norepinephrine and normetanephrine has increased significantly, suggesting that salt loads may enhance sympathetic nerve function in normal mice.	[42]
2- Reproductive functions of male	Merino rams	12% NaCl diet	Smaller testes, reductions in spermatogenesis, lower ejaculate volumes, and decreased sperm concentration. In vitro fertilization, hatching rate was lower for sperm from rams on the high-salt diet. Reduced the plasma levels of sex (FSH, LH, and T) and metabolic (insulin and leptin) hormones. Decreased mRNA expression of sex hormone receptors (CYP17A1, CYP11A1, FSHR, LHR, and AR), as well as major spermatogenesis genes (Cyclin A, Hsp70, and c-kit).	[3]
	Rats	8% salt diet	The viability, concentration, morphology, and motility of sperm have been changed substantially. The serum level of testosterone was significantly reduced. Salt intake resulted in an increase in serum corticosterone concentration. MDA level was significantly increased in salt fed rats. In treated rats, SOD was significantly improved compared with control.	[24]
	Rats	8% salt diet	Increased sperm count was observed in the high salt diet-treated rats. Increased abnormal sperm cells and increased epididymal oxidative stress. In high salt diet-treated rats, FSH and testosterone concentrations increased, whereas LH concentrations decreased.	[37]
	Golden spiny mice	5%	The osmolarity of urine exhibited a remarkable rise in salinity below 5 percent. Testis mass and spermatogenesis have decreased considerably. Increased salinity has substantially reduced body weight. Recovering subjects quickly regained their body weight and after only four weeks they exceeded initial values. Testis weight and spermatogenesis, however, showed no recovery.	[32]
	Common spiny mice	3.5%	The osmolarity of the urine revealed a significant rise of less than 3.5 percent. Increase in salinity did not reduce body mass in *A. cahirinus*. Recovering subjects quickly regained their body weight and after only four weeks they exceeded initial values. Testis weight and spermatogenesis, however, showed no recovery.	[32]
3- Reproductive functions of female	Mouse	4% NaCl water	Prevented follicle growth by causing theca and granulosa cells apoptosis and suppressed granulosa cell proliferation.	[7]
	Merino ewes	(NaCl 13% of dry matter)	Lower levels of insulin and leptin. During gestation, the concentration of T3 varied, leading to lower levels in the high-salt group during the first third of pregnancy and higher levels in the final third of pregnancy. In the first two-thirds of pregnancy, the concentration of T4 was less in ewes eating high-salt diet. No significant effects of high salt consumption have been observed on the rates of pregnancy, weights of lamb birth, or composition of milk.	[22]
